# Blocking Estrogen Synthesis Leads to Different Hormonal Responses in Canine and Human Triple Negative Inflammatory Breast Cancer

**DOI:** 10.3390/cancers13194967

**Published:** 2021-10-02

**Authors:** Sara Caceres, Beatriz Monsalve, Angela Alonso-Diez, Belén Crespo, Maria Jose Illera, Paloma Jimena de Andres, Gema Silvan, Juan Carlos Illera

**Affiliations:** 1 Department of Animal Physiology, Veterinary Medicine School, Complutense University of Madrid (UCM), 28040 Madrid, Spain; b.monsalve@ucm.es (B.M.); belencre@ucm.es (B.C.); mjillera@ucm.es (M.J.I.); gsilvang@ucm.es (G.S.); jcillera@ucm.es (J.C.I.); 2 Department of Animal Medicine, Surgery and Pathology, Veterinary Medicine School, Complutense University of Madrid (UCM), 28040 Madrid, Spain; angalo02@ucm.es (A.A.-D.); pjdeandres@vet.ucm.es (P.J.d.A.)

**Keywords:** estrogen, aromatase inhibitors, STS inhibitors, inflammatory breast cancer

## Abstract

**Simple Summary:**

Estrogen is responsible for tumor progression, and blocking its synthesis is effective in certain breast cancers. Therefore, the aim of this study is to determine the effect of letrozole (anti-aromatase) and STX-64 (anti-sulfatase) in canine and human inflammatory breast cancer cell lines and xenografts. The results reveal that letrozole blocks estrogen synthesis, reducing tumor progression. However, STX-64 increases estradiol synthesis, increasing tumor progression. In summary, letrozole may be an effective treatment for canine and human inflammatory breast cancer.

**Abstract:**

Blocking estrogen synthesis by inhibitors of estrogen synthesis is a widely used therapy against estrogen receptor-positive tumors. However, these therapies are less effective in negative expression tumors. Therefore, this study determined the effectiveness of anti-aromatase and anti-sulfatase therapies in canine and human inflammatory breast cancer. Cell cultures and xenografts from IPC-366 and SUM149 were treated with different doses of letrozole (anti-aromatase) and STX-64 (anti-sulfatase), in order to observe their effectiveness in terms of cell proliferation, tumor progression, and the appearance of metastases and hormonal profiles. The results revealed that both treatments are effective in vitro since they reduce cell proliferation and decrease the secreted estrogen levels. In xenograft mice, while treatment with letrozole reduces tumor progression by 30–40%, STX-64 increases tumor progression by 20%. The hormonal results obtained determined that STX-64 produced an increase in circulating and intratumoral levels of estradiol, which led to an increase in tumor progression. However, letrozole was able to block estrogen synthesis by decreasing the levels of circulating and intratumoral estrogen and thus slowing down tumor progression. In conclusion, letrozole can be an effective treatment for canine and human inflammatory breast cancer. The knowledge of the hormonal profile of breast tumors reflects useful information on the effectiveness of different endocrine treatments.

## 1. Introduction

Estrogen has long been described as a key regulator of breast cancer growth and differentiation. Approximately 75% of breast cancers are estrogen receptor (ER)-positive [1]. Therefore, treatments against the synthesis or action of estrogen have been extensively used.

Two principal pathways are implicated in the estrogen formation in breast cancer tissues: the aromatase pathway, mediated by the enzyme CYP19-aromatase, which transforms androgen into estrogen, and the sulfatase pathway, which converts estrone into estrone sulfate by the enzyme sulfotransferase [2]. 

In post-menopausal women, there are two sources of estrogen. Estrogen may arise from aromatase activity in extraovarian body sites, such as subcutaneous adipose tissue and skin, and may reach breast cancer in an endocrine manner. On the other hand, an increase of local concentrations of estrogen may result from aromatase overexpression within the tumor tissue [1,3]. 

Tamoxifen is the first-line treatment of endocrine-sensitive breast cancer, although it presents several side effects [1,4]. In the last years, the most effective hormonal treatment of post-menopausal patients with ER-positive breast cancer has been the use of aromatase inhibitors such as letrozole. These treatments block aromatase activity in the breast and peripheral tissues, thereby reducing the amount of local estrogen production, which in turn helps to suppress the recurrence of the breast tumor tissue [3,4,5]. Other effective treatments, which target estrogen synthesis, are the steroid sulfatase (STS) inhibitors. STX-64 is a first-generation treatment that is shown to inhibit mammary tumor growth [6]. These endocrine therapies are well established as adjuvant therapies for postmenopausal women with positivity to hormone receptors. Treatments with aromatase inhibitors improved significantly the 5-years disease-free survival compared to treatments like tamoxifen and have less adverse effects compared to tamoxifen [7]. Regarding STS inhibitors, few clinical trials have been developed, but all of them agree that it is a well-tolerated treatment that can be used in patients with ER + breast cancer. In addition, clinical trials have been conducted combining aromatase inhibitors and STS inhibitors reporting clinical benefits in ER+ patients [8]. 

However, these treatments have a poor effect on triple negative breast cancer (TNBC). This subtype of breast cancer is characterized by a lack of estrogen receptor, progesterone receptor, and HER-2 and accounts for around 15% of breast cancers [9]. Despite its negativity to receptor expression, several TNBC tumors are able to synthetize steroid hormones locally that regulate tumor growth and progression, such as canine and human inflammatory breast cancer tumors [10,11]. Inflammatory breast cancer (IBC) in woman and inflammatory mammary cancer (IMC) in dogs represent a subtype of one of the worst mammary tumors, which accounts for 6% of diagnosed breast cancers and presents a poor survival rate [10,11,12]. Comparative oncological studies had proposed the use of IMC tumors as a model for human disease research, due to its similarities in terms of clinical, molecular, and histological aspects in both species [10,11]. It has been shown that ER+ IBC tumors had a lower response to endocrine therapy, indicating that IBC may present endocrine therapy resistance [13]. 

Therefore, this study aims to investigate the effect of endocrine-disrupting treatments (aromatase and STS inhibitors) on estrogen synthesis in cell cultures and xenografts from IBC and IMC cell lines (SUM149 and IPC-366), in order to determine cell viability, tumor progression, and the hormonal changes produced by the treatments and thus elucidate the efficacy of these treatments in IBC and IMC.

## 2. Materials and Methods

### 2.1. Inflammatory Breast Cancer Cell Lines

IPC-366 was obtained from the Department of Physiology of the Veterinary Medicine School (University Complutense of Madrid, Madrid, Spain), and was cultured in Dulbecco’s modified Eagle medium nutrient mixture F-12 Ham (DMEM/F12) containing 5% charcoal stripped FBS (Sigma Aldrich, Madrid, Spain), 1% penicillin-streptomycin solution, and 1% L-glutamine (Sigma Aldrich). 

SUM149 was obtained from Asterand, plc (Detroit, MI, USA) and maintained in Ham’s F-12 media supplemented with 5% charcoal stripped FBS (Sigma Aldrich), 1 µg/mL hydrocortisone, 5 µg/mL insulin, and 1% penicillin-streptomycin solution (Sigma Aldrich, Madrid, Spain). 

The cells were cultured in 25 cm^2^ culture flasks and were maintained in a humidified atmosphere of 5% carbon dioxide at 37 °C. The cell cultures were observed daily by a phase-contrast microscopy to check cell viability and growth.

### 2.2. Treatments

Letrozole, an aromatase inhibitor, and STX-64, a STS inhibitor, were obtained from Sigma Aldrich (Madrid, Spain). All compounds were dissolved in DMSO, stored at −20 °C, and diluted in fresh culture media immediately before use. In order to determine the final concentrations to be used of letrozole and STX-64, sensitivity assays were carried out in IPC-366 and SUM149 cells with different concentrations of the compounds. Cultured IPC-366 and SUM149 cells were divided into a control group (a final concentration of DMSO < 0.1%) and experimental groups with 3 different doses of the compounds (letrozole: 0.35, 0.7, and 1.04 μM; STX-64: 1.5, 3.0, and 4.5 μM).

### 2.3. Cell Viability Assay (MTS Assay)

IPC-366 and SUM149 cells were cultured in 96-well plates and treated with letrozole and STX-64 doses for 24, 48, and 72 h. For each time-point, culture media from the control and experimental groups were collected, and cells were assayed for cell viability using the CellTiter 96R Aqueous One Solution Cell Proliferation Assay according to the manufacturer’s instructions (Promega, Madrid, Spain). Briefly, MTS was added to culture media on each well and incubated for 3 h at 37 °C, 5% CO_2_. Absorbance was then read at 490 nm with a 96-well SpectraMax 190 UV/Vis plate reader. Untreated cells were taken to represent 100% proliferation, and that all the drug-treated cells were expressed relative to this.

### 2.4. Experimental Animals and Treatment

Eighty-four female Balb/SCID mice were obtained from Janvier Labs (Madrid, Spain), early in the morning with dams to minimize shipping stress and adapted for 7 days in the Animal Facility (Department of Animal Physiology, School of Veterinary Medicine, University Complutense of Madrid). The mice were housed in polycarbonate cages and used after they were acclimated in an environmentally controlled room (temperature: 23 ± 2 °C, relative humidity: 50 ± 10%, frequent ventilation, and a 12 h light cycle). The mice were fed with soy-free pellet food (Dyets Inc., Bethlehem, PA, USA). The required sample size needed to compare the normal means of the seven experimental groups simultaneously (control plus three treatments of letrozole and three treatments of STX-64) was performed using the sample size determination module of the statistical package Statgraphics Centurion XVI (Statpoint Technologies, Inc., Warrenton, VA, USA). The Institutional Animal Care and Use Committee of the University Complutense of Madrid, Spain, approved the experimental protocols for this study (number: Proex 31/15). All the procedures were completed in accordance with the Guide for the Care and Use of Laboratory Animals and conformed to the relevant EU Directive.

A suspension of 106 IPC-366 and SUM149 cells were subcutaneously inoculated in the ventral region of 6–8-week-old female Balb/SCID mice. The mice were inspected weekly for the development of tumors. Tumor volumes were determined every 3 days by measuring the length and width and then calculating using the following formula: volume = (width)/2 × (length)/2 [14]. When tumors were detected and reached a volume of 0.5 cm^3^, mice were injected subcutaneously every 3 days with different doses of letrozole and STX-64 for a total of 15 days. Animals were divided in 7 groups: a control group (n = 12; 6 of IPC-366 and 6 of SUM149), where mice were injected with PBS, and 6 experimental groups (n = 12 each group; 6 of IPC-366 and 6 of SUM149) that were treated with a dosage of 1, 5, and 10 mg/kg of letrozole and STX-64, respectively. Letrozole and STX-64 doses were chosen based on the results described in the literature [15,16]. Mice were sacrificed when tumors reached a volume of 1.5 cm3 or at the end of treatment. At this point, animals were anaesthetized with isoflurane (IsoVet) at 4% for induction and 1.5% for maintaining sedation, supplied in a fresh gas flow rate of 0.5 L of oxygen/minute, and blood samples were obtained intracardially. Animals were then sacrificed by a lethal dose of isoflurane. Tumors were harvested at necropsy for homogenates, and the apparition of metastases in lungs and liver was determined at necropsy macroscopically.

Tumors were homogenized in PBS (pH 7.2) and centrifuged at 1200× *g*, for 20 min at 4 °C. Supernatants were collected and stored at −20 °C until hormone assays. Blood samples were centrifuged at 1200× *g* and 4 °C for 20 min, and serum was separated and stored at −20 °C until assayed.

### 2.5. Steroid Determinations in Culture Media, Serum, and Tumor Homogenates

The hormones determined and the antibodies used in this study are summarized in Table 1. Progesterone (P4), androstenedione (A4), testosterone (T), estrone sulphate (E1SO4), and 17β-estradiol (E2) antibodies were developed in the Department of Animal Physiology (UCM, Spain). To determine these hormones in tumor homogenate samples, a previously validated competitive validated enzyme-immunoassay (EIA) was carried out [17]. For serum samples and culture media samples, an amplified EIA previously validated was performed [18]. The technical procedure of the EIA assays has been previously described [9]. Pregnenolone (P5), dehydroepiandrosterone (DHEA), and dehydrotestosterone (DHT) determinations were performed using a commercially available EIA kit (Demeditech, Germany) according to the manufacturer’s instructions. These kits presented cross reactivity to canine and human steroids.

All the hormone concentrations are expressed in ng/g (for tumor homogenates) and ng/mL (for serum samples and culture media), except the DHT culture medium concentrations, which are expressed in pg/mL.

### 2.6. Statistics

The Kolmogorov–Smirnoff test was used to assess the goodness-of-fit distribution of the data. For cell viability and tumor growth assays, differences between the control and experimental groups were analyzed by one-way analysis of variance (ANOVA). As hormonal determination data was nonparametric, for comparison between the control and treatment groups of both cell lines, the Mann–Whitney (Wilcoxon) W test was performed using SAS 9.4. Data are shown as means ± standard deviation. In all statistical comparisons, *p* values < 0.05 were considered statistically significant.

## 3. Results

### 3.1. Deprivation of Estrogen Production Produced an Anti-Proliferative Effect in Cultured Conditions

**Figure 1:**
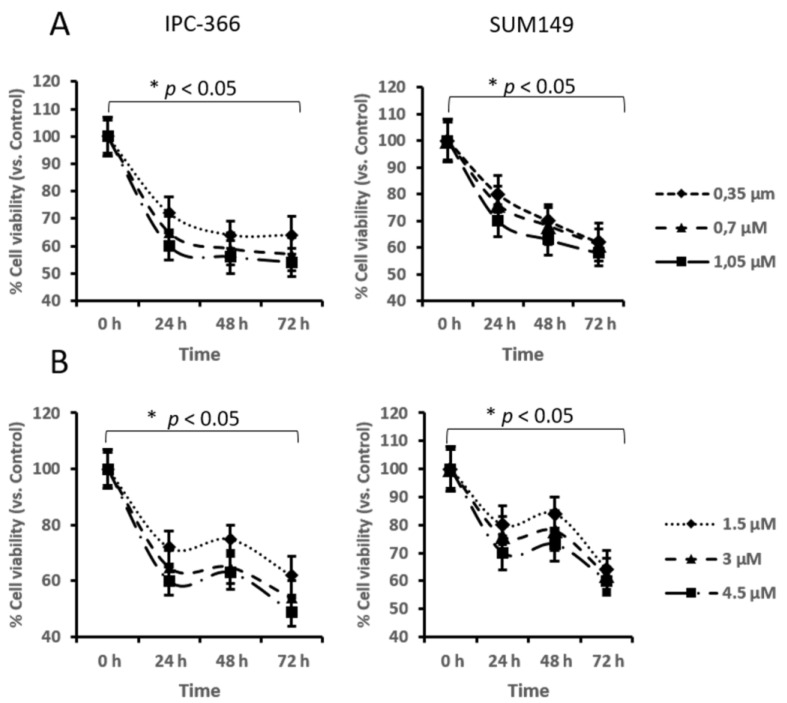
MTT assay results from letrozole (**A**) and STX (**B**) treatments from IPC-366 and SUM149 culture cells. * denoted significant differences (*p* < 0.05) between control and treatments.

Letrozole and STX64 treatments in IPC-366 and SUM149 cultured cells promoted a significant dose-dependent decrease (*p* < 0.05) at all the doses studied. Letrozole reduced cell viability in approximately 40–50%, while STX-64 decreased cell viability by around 40% in both cell lines (Figure 1). STX-64 in vitro treatment resulted in a slight augmentation of cell viability at 48 h, although it decreased at 72 h (Figure 1B).

### 3.2. Inhibition of Estrogen Production Promotes Androgen Secretion in Cultured Conditions

**Figure 2:**
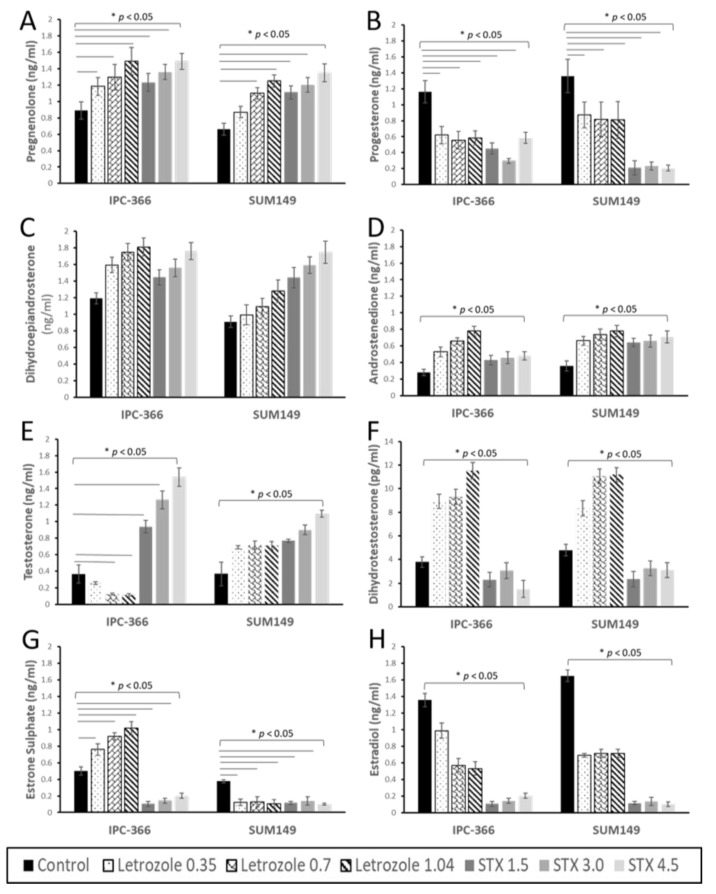
Steroid hormone concentrations of (**A**) P5; (**B**) P4; (**C**) DHEA; (**D**) A4; (**E**) T; (**F**) DHT; (**G**) E1SO4 and (**H**) E2 in culture media of IPC-366 and SUM149 cells treated with different doses of letrozole and STX-64. Culture media was collected 72h after treatment. * denoted significant differences (*p* < 0.05) between control and treatments.

Steroid hormone (P5, P4, A4, DHEA, T, DHT, SO4E1, and E2) concentrations of IPC-366 and SUM149 were measured in culture media after letrozole and STX-64 treatments (Figure 2). Similar hormonal responses were found in IPC-366 and SUM149 cell lines. Results revealed that levels of precursor steroid P5 increased significantly (*p* < 0.05) after letrozole and STX-64 additions, and P4 concentrations decreased significantly (*p* < 0.05) with respect to the control (Figure 2A,B). Regarding androgen precursor secretion, DHEA and A4 levels were significantly increased (*p* < 0.05) in both treatments and cell lines (Figure 2C,D). However, several differences were found in T and DHT secretion between treatments and cell lines (Figure 2E,F). Although T levels increased significantly (*p* < 0.05) in the presence of letrozole and STX-64 in SUM149, in the canine cell line IPC-366, letrozole treatment resulted in decreased T levels, while STX-64 promoted a significant (*p* < 0.05) increase in T secretion (Figure 2E). Indeed, DHT levels were augmented significantly (*p* < 0.05) in letrozole treatment but diminished significantly (*p* < 0.05) when STX-64 treatment was added (Figure 2F). 

Estrogen concentration results showed that E2 levels in both treatments and cell lines decreased significantly (*p* < 0.05). Though letrozole and STX-64 treatments are thought to compromise estrogen production, in IPC-366, E1SO4 levels were increased significantly (*p* < 0.05) after letrozole treatment, while they were decreased in SUM149. However, E1SO4 concentrations diminished significantly (*p* < 0.05) in addition to STX-64 treatment in both cell lines (Figure 2G,H). 

### 3.3. Letrozole Treatment Reduced Tumor Progression While STX-64 Promoted Tumor Progression

**Figure 3:**
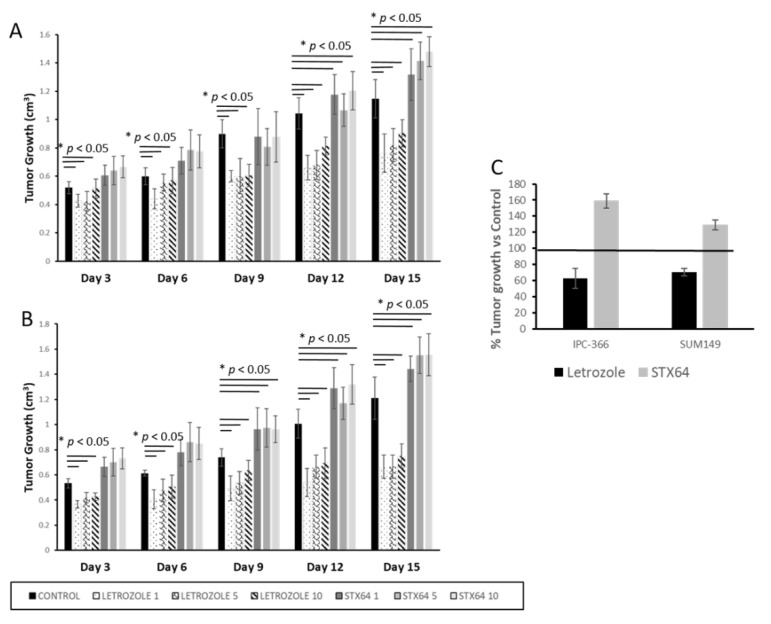
Percentage of tumor growth in SCID mice inoculated with (**A**) IPC-366 and (**B**) SUM149 cells. Animals were treated with 1, 5 and 10 mg of letrozole or STX-64 during 15 days. Bar represents average percentage ± SD. * denoted significant differences between control and treatments. (**C**) Global representation of the effect of letrozole and STX-64 treatment on IPC-366 and SUM149 tumors. Bars denoted the mean percentage of tumor growth of all letrozole and STX64 treated mice versus control group at the end of the experiment. Black line represents control group as 100%. Letrozole treatment reduced approximately 30% of tumor growth, while STX-64 augmented tumor volume around 20%.

The effect of letrozole and STX64 treatments on tumor progression in xenografts from IPC-366 and SUM149 is represented in Figure 3. Results revealed that letrozole treatment provoked a significant decrease (*p* < 0.05) in tumor progression after the third day of treatment. Interestingly, low doses of letrozole (1 mg/kg) produced the highest reduction on xenografts from both cell lines (Figure 3A). In addition, STX-64 treatment significantly increased (*p* < 0.05) tumor progression from the ninth day of treatment (Figure 3B).

Therefore, as letrozole treatment reduced tumor progression by approximately 30–40%, STX-64 treatment increased tumor progression by approximately 20% in both cell lines (Figure 3C). 

Remarkably, STX-64 treatment on xenograft mice from IPC-366 and SUM149 resulted in the absence of any apparition of metastasis in lung and liver. However, letrozole treatment significantly reduced (*p* < 0.05) the apparition of metastases in lung and liver in xenografts from both cell lines (Table 2).

### 3.4. Letrozole and STX-64 Induced Different Intratumoral and Circulating Hormonal Responses in Xenograft Mice

**Figure 4:**
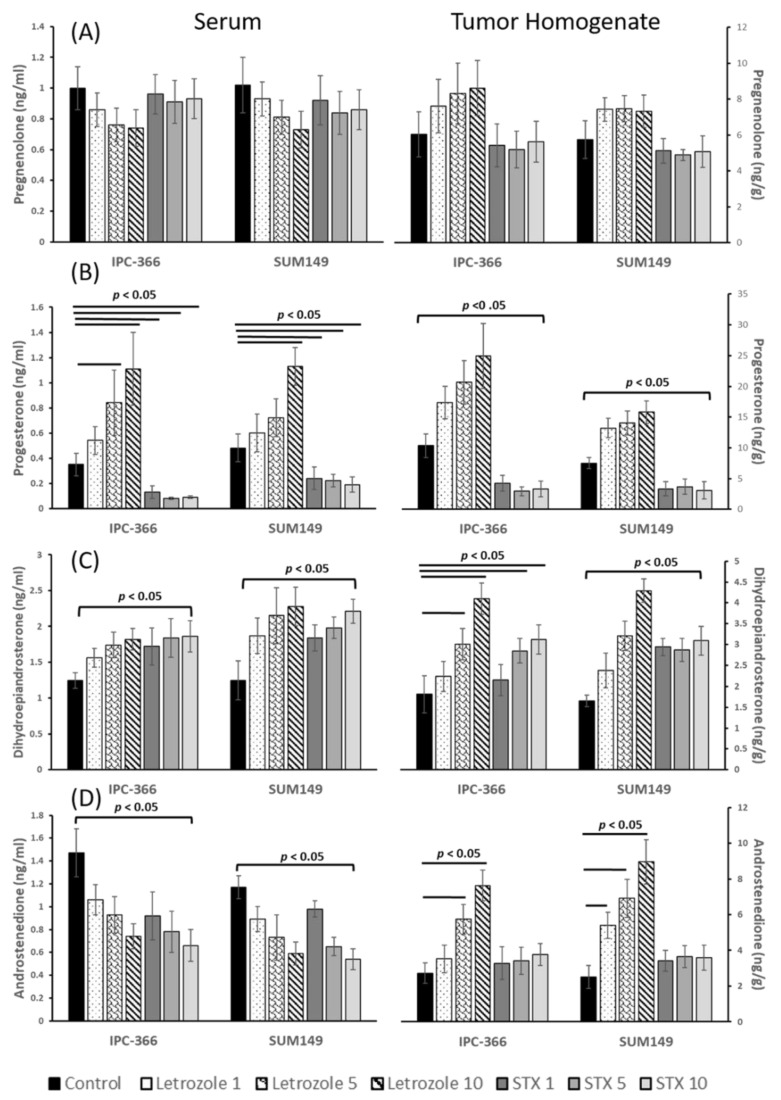
Steroid concentrations of (**A**) Pregnenolone (P5); (**B**) Progesterone (P4); (**C**) Dehydroepiandrosterone (DHEA) and (**D**) Androstenedione (A4), in serum and tumor homogenate samples of treated xenografts from IPC-366 and SUM149. Results showed an increased in DHEA concentrations on serum and tumor samples of xenografts treated with letrozole and STX-64. P4 levels increased in mice treated with all doses of letrozole and decreased in mice treated with STX-64. Bar represents means ± SD. significant differences between control and treated groups (*p* < 0.05).

**Figure 5:**
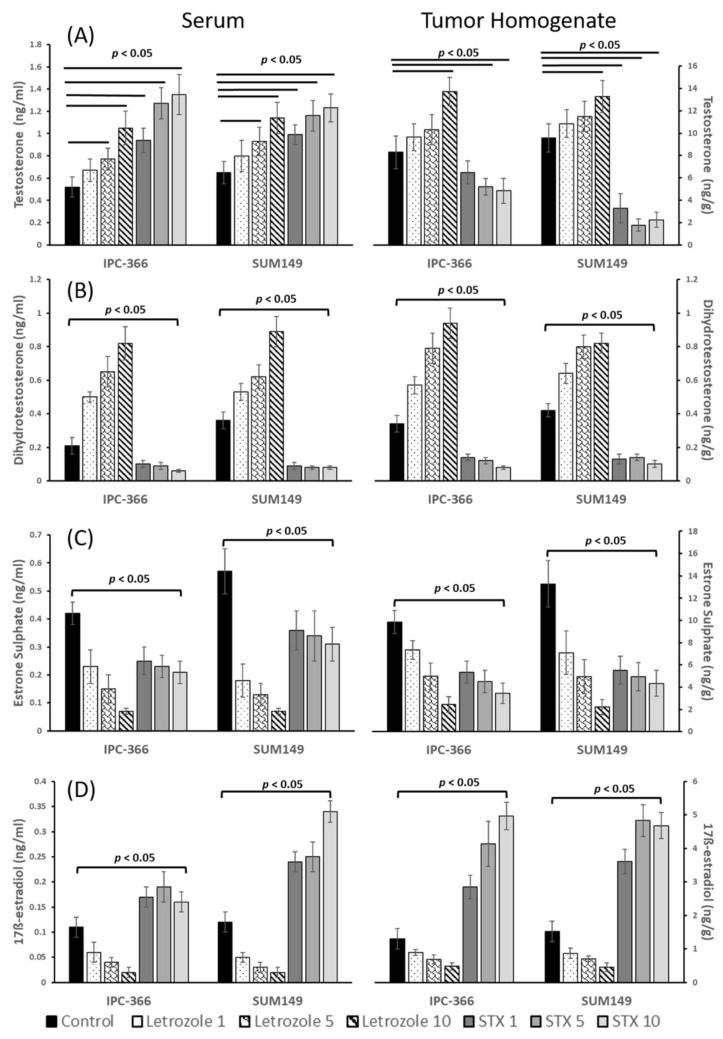
Steroid concentrations of (**A**) Testosterone (T); (**B**) Dehydrotestosterone (DHT); (**C**) Oestrone sulphate (SO4E1) and (**D**) 17β-oestradiol (E2 in serum and tumor homogenate samples of treated xenografts from IPC-366 and SUM149. Results showed an increased in androgen concentrations and a decrease in estrogen concentrations on serum and tumor samples of xenografts treated with letrozole and STX-64. Bar represents means ± SD. significant differences between control and treated groups (*p* < 0.05).

Results from steroid hormone assays on tumor homogenates and serum samples from IPC-366 and SUM149 xenografts are summarized in Figure 4 and Figure 5. Both xenografts showed a similar hormonal response after treatments.

Results from serum and tumor homogenate P5 concentrations showed no significant differences after letrozole and STX-64 treatment (Figure 4A). Regarding P4 concentrations, differences were found between treatments. While letrozole provoked a significant increase (*p* < 0.05) in serum and tumor homogenate P4 levels, STX-64 significantly reduced (*p* < 0.05) these levels. However, both treatments produced a significant increase (*p* < 0.05) in DHEA concentrations in serum and tumor homogenate samples (Figure 4B,C).

Interestingly, in A4 concentrations, a significant decrease (*p* < 0.05) was found in serum samples from letrozole- and STX-64-treated mice, but in tumor homogenates, these levels increased significantly (*p* < 0.05) after letrozole treatment and remained after STX-64 treatment (Figure 4D). 

Results from androgen determinations revealed that letrozole induced a significant increase (*p* < 0.05) in T and DHT levels in serum and tumor homogenate samples. Contrarily, STX-64 provoked a decrease in T and DHT concentrations, except for serum T levels, which were augmented (Figure 5A,B). 

Furthermore, both treatments significantly reduced (*p* < 0.05) serum and tumor homogenate E1SO4 levels, this decline being greater after letrozole treatment. However, STX-64 treatment produced a significant elevation (*p* < 0.05) in serum and tumor homogenate E2 levels, while letrozole treatment decreased them significantly (*p* < 0.05) (Figure 5C,D).

## 4. Discussion

Some authors consider normal and neoplastic glands as an endocrine tissue due to their capability for synthesizing estrogen and androgen [19,20]. Recently, it has been reported that canine and human mammary tumors are able to produce steroid hormones [10,11]. Moreover, under culture conditions, neoplastic cells are also capable of secreting steroid hormones [18].

Estrogen has long been suspected as the hormone responsible for increasing breast cancer risk and breast tumor progression [5,21]. Endocrine therapies aimed at inhibiting the synthesis or action of estrogen, either by blocking estrogen production or ER, are first-line and successful therapies for many women with ER-positive breast cancer. However, these treatments present limitations since their administration can produce important side effects and does not exempt the risk of recurrences [1]. Such endocrine therapies are known to be insensitive on triple negative breast cancers, although new approaches have denoted that these patients could benefit from these treatments, as compounds such as tamoxifen could act through an ER-independent pathway [4]. 

Given the evidence that canine and human inflammatory breast cancer have an important hormonal influence [10,11], the use of therapies that block estrogen synthesis may be an approach as future therapeutic targets for this type of cancer. Therefore, in this study, we evaluated the effect of letrozole (aromatase enzyme inhibitor) and STX-64 (STS enzyme inhibitor) on the hormonal response of cell cultures and xenografts from IPC-366 and SUM149 cell lines.

All endogenous steroids are derived from cholesterol and are synthetized in the adrenal gland and ovaries [2]. Cholesterol is converted to P5 by the P450scc enzyme, which is the precursor for other steroid hormones. P5 leads to the production of DHEA and P4, by two different pathways catalyzed by different enzymes [22]. In the late 1970s, Abul-Hajj and collaborators revealed that most of the ER-tumors had the required enzymes to convert P5 into active estrogen and androgen and postulated that this capacity could be attributed to their failure to respond to endocrine therapies [23]. Nowadays, P5 has been proposed as a candidate for hormonal therapy due to its relation to the ERβ signaling pathways [24]. 

Recently, it has been shown that the addition of compounds such as flutamide to breast cancer culture cells increased secreted P5 levels [25]. In accordance with this, we found that letrozole and STX-64 treatments increased secreted P5 levels in human and canine inflammatory breast cancer cell lines. Taking into account that letrozole and STX-64, as well as flutamide, disrupt hormone synthesis at different levels, it can be assumed that the administration of these compounds produces a change in steroid synthesis that begins with a high secretion of P5 by the neoplastic cells.

These results can be related to the reduction of cell viability when cells are treated with letrozole or STX-64. In both cases, the reduction in cell viability did not exceed 50%. Therefore, neoplastic cells are capable of adapting to different hormonal environments, producing different hormones de novo depending on the cellular and survival needs.

Assuming that the increase in secreted P5 produces a dysregulation of hormonal synthesis, we observe that, when we block estrogen synthesis with letrozole or STX-64, there is a decrease in secreted levels of P4 and an increase in DHEA levels. Surprisingly, previous studies with androgen receptor inhibitors demonstrated that the opposite occurred; in cultured conditions, secreted levels of DHEA increased, and those of P4 decreased [25]. These results may translate into a deprivation of estrogen synthesis in neoplastic cells, which will lead to the consumption of P4 for their estrogen production, while blocking the action of androgen promotes its synthesis via DHEA. Regardless of the synthesis pathway, we observed that anti-estrogenic treatments in breast cancer cells produce an increase in secreted A4 levels. A4 is the main precursor either for androgen (A4 converts to T through 17βHSD activity, which converts to DHT) or estrogen (A4 converts to E1 through aromatase activity, which converts to E2) [2]. Therefore, high levels of secreted A4 support the idea that neoplastic cells still produce steroid hormones under anti-estrogenic treatments in order to survive.

It is known that estrogen promotes cell proliferation and tumor progression in breast cancer [1]. However, there is an actual controversy in the role of androgen in breast cancer. While some authors postulated a protective and anti-proliferative role of androgen in breast cancer [26], others suggested that androgen could exert a proliferative effect [27]. 

**Figure 6:**
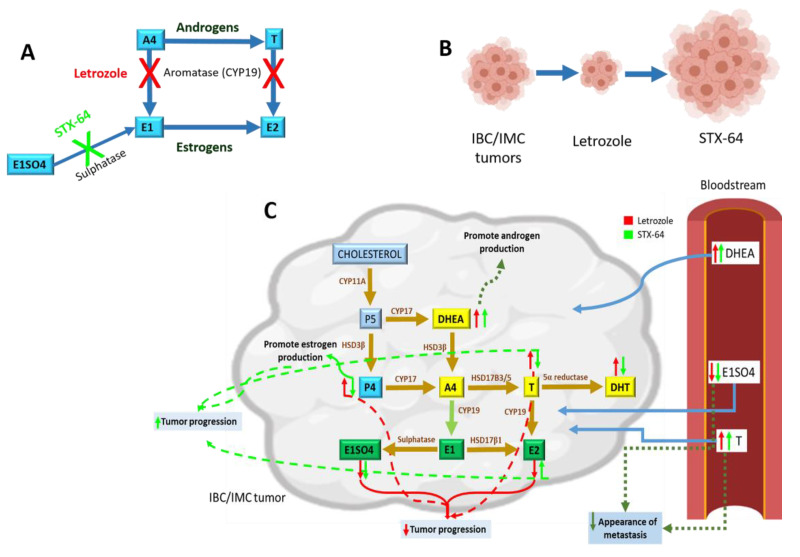
Scheme of the effect of estrogen blockade on steroid hormone synthesis. (**A**) Routes of estrogen synthesis from androgens highlighting the effect of letrozole as an aromatase inhibitor, and STX-64 as an estrone sulfatase inhibitor. (**B**) Represents the control tumors of the IPC-366 and SUM149 xenografts, and the effect of letrozole on them, which decreases tumor progression, and of STX-64, which increases tumor progression. (**C**) Effect of letrozole and STX-64 treatments on the steroidogenic pathways in tumors and serum from IPC-366 and SUM49 xenografts. Briefly, an intratumoral increase in DHEA levels has been shown to promote androgen synthesis, while a decrease in P4 promotes estrogen synthesis. High levels of intratumoral estrogens are related to an increase in tumor progression. Additionally, high levels of circulating testosterone in addition to low levels of E1SO4 are related to a low appearance of metastasis.

In this study, different hormonal responses, in terms of androgen and estrogen secretion, were found when cells were treated with letrozole and STX-64 (Figure 6A). Letrozole is an aromatase inhibitor that reduces cell proliferation and growth in breast cancer cell lines and xenograft models [5]. It has been shown that the use of aromatase inhibitors in patients with breast cancer as adjuvant therapy reduces the 10-year mortality in 40% of cases. However, there are patients that acquire resistance to these therapies [28]. On the other hand, STX-64 is a first-generation STS inhibitor that showed promising results in the treatment of advanced breast cancer [29]. Taking into account the action of letrozole and STX-64 as estrogen-depriving treatments, our results showed that secreted estrogen levels (E2 and E1SO4) decrease after both treatments, as described by other authors [6,16]. The decrease in estrogen levels could be involved in the reduction of cell viability.

Interestingly, in IPC-366 cells treated with letrozole, secreted E1SO4 levels increased. E1SO4 has been reported to act as a reservoir of estrogen for neoplastic cells [25,30]. Some authors suggested that E1SO4 contributes to aromatase inhibitor resistance in breast cancer cell lines [31]. Therefore, IPC-366 may present resistance to aromatase inhibitors, but further studies are needed to determine this. 

Indeed, the letrozole treatment results revealed that secreted androgen levels (T and DHT) were higher than the control group. A possible explanation is the accumulation of androgen, due to the deprivation of estrogen synthesis that could contribute to the anti-proliferative effect of letrozole. Surprisingly, IPC-366 showed a decrease in T levels. T probably exerts its action through its union to the androgen receptor (AR). AR is expressed in 15–30% of TNBC, and some studies have suggested that AR might function in place of ER in its absence [32]. Contrarily, when cells were treated with STX-64, DHT secretions were significantly diminished. These results are in line with other authors that obtained similar results [6]. This decrease in DHT levels could be responsible for the poor response of STX-64 in culture conditions.

Regarding letrozole- and STX-64-treated mice, results revealed that letrozole treatment reduced tumor progression by approximately 30–40% in IPC-366 and SUM149 xenografts, according to other authors [5,33]. However, STX-64 treatment promotes tumor progression, leading to an augmented tumor volume of around 20%, indicating that this treatment is not effective on IMC and IBC (Figure 6B). Other authors that evaluated STX-64 efficiency in MCF-7 xenografts revealed that this treatment did not show significant differences in tumor volumes, determining that STX-64 attenuated tumor growth [34]. The fact that we found an increase in tumor progression in the case of the STX-64 may be partly due to changes in the regulation of hormone secretion. 

In view of the results obtained in the circulating and intratumoral hormonal levels of the steroid hormones studied in the mice treated with letrozole and STX-64, hormonal dysregulation was observed at all levels of the steroidogenic pathway, which promoted or inhibited tumor progression (Figure 6C). First, an increase in DHEA levels was observed after both treatments. As in cultured media, DHEA may promote androgen production. Furthermore, in in vivo systems, the increase in both circulating and intratumoral DHEA may be due to the fact that, in the presence of hormone disruption treatment, other peripheral tissues capable of producing hormones may be affected. Therefore, the adrenal gland, the major producer of DHEA, produces large amounts of this hormone, which pass into the peripheral bloodstream until it reaches the peripheral tissues, exerting its action and promoting hormonal synthesis.

It is known that estrogen and P4 are involved in cell proliferation, although the mechanism of action is still unclear. On the contrary, it has also been described that high levels of progesterone in the normal breast epithelium are associated with a decrease in mitotic activity [35]. In this study, results revealed differences in P4 serum and intratumoral concentrations after treatments. Mice treated with letrozole showed high P4 levels, while mice treated with STX-64 showed a decrease in P4 levels. Assuming that high levels of P4 could be related to a decrease in mitotic activity, our results are in line with this, as mice treated with letrozole showed tumor growth reduction and high P4 levels, and mice treated with STX-64 showed tumor progression and low P4 levels, which can be consumed to promote estrogen synthesis. 

Results from estrogen and androgen concentrations from mice treated with letrozole are in line with other authors [36]. In this study, androgen concentrations increased while estrogen concentrations decreased. Upon inhibiting aromatase activity, androgen was accumulated, as it could not be converted to estrogen, so its levels decreased. Therefore, the low levels of estrogen caused by the effect of letrozole promote a slowdown in tumor progression, indicating that letrozole treatment is effective in order to maintain low estrogen levels and elicit good treatment responses. 

Regarding STX-64 treatment, interesting results from estrogen and androgen concentrations were found. By inhibiting STS activity, circulating and intratumoral E2 levels increased. This might be associated with the increase in tumor progression found in treated mice. Despite the negative expression of ER in IPC-366 and SUM149, the elevation of E2 levels found in this treatment, and its association with an increase in progression, make us consider that E2 is acting through mechanisms independent of ER or through ERβ signaling pathways. Other authors support this claim since they described that E2 can influence the development of breast cancer through mechanisms independent of ER [37]. However, these mechanisms are poorly studied, and more studies are needed at the genetic level to elucidate the role of E2 in triple negative breast cancer.

It has also been reported that T could be associated with tumor progression. High intratumoral T levels can inhibit tumor progression in IBC and IMC xenografts [25]. Our results are in line with these, as significantly low concentrations of intratumoral T were found in mice treated with STX-64, indicating that high intratumoral E2 levels and low T levels are involved in tumor progression. The inhibition of STS probably leads to an increase of aromatase activity that promotes low levels of T, which is consumed to produce E2 and raise E2 levels. None of the STX-treated mice had distant metastasis, and low E1SO4 levels were found. Some authors have observed an association between estrone sulfatase expression and the presence of lymph node metastases [38]. Therefore, even though STX-64 failed to be an effective treatment for IBC and IMC, the inhibition of estrogen sulfation prevents its accumulation and transfer to the bloodstream, reducing the probability of appearance of metastases and giving importance to E1SO4 levels as a prognostic value. Moreover, it has recently been shown that high circulating levels could be related to a lower probability of appearance of metastasis [10,25], which is in accordance with our results, which revealed that mice treated with STX-64 showed an increase in T serum concentrations. 

In summary, letrozole and STX-64 treatments block estrogen synthesis at different levels and produce different effects. Letrozole, by blocking aromatase activity, decreases E2 levels, producing a reduction in tumor progression. On the other hand, STX-64 produces an increase in E2 and a decrease in intratumoral T levels, which promotes tumor progression. However, taken together, the decrease in E1SO4 and the increase in T circulating levels promote a low appearance of metastasis.

## 5. Conclusions

Letrozole is an effective treatment for IBC and IMC since it reduces estrogen levels, although STX-64 is not a recommended treatment for this type of cancer since, despite inhibiting estrogen sulfation, it increases E2 levels. It has also been shown that neoplastic cells are able to adapt to different hormonal environments and produce hormones de novo in order to survive. The importance of E1SO4 in tumor progression and the appearance of metastasis have also been demonstrated. However, further studies are needed in order to determine the role of E1SO4 and the adaptation mechanisms of neoplastic cells. Therefore, blocking the synthesis of estrogen may present different effects that depend on whether neoplastic cells can synthetize steroid hormones, so knowledge regarding both the hormonal profiles and the response to various endocrine treatments is crucial to understand the progress of the disease and the possible physiological consequences of the treatments used.

## Figures and Tables

**Table 1:** Steroid hormones assayed and antibodies used for EIA determinations. P5, DHEA and DHT were determined using commercial kits following manufacturer’s instructions.

Hormone	Abbreviation	Antibody Code	Dilution
Progesterone	P4	C914	1/6000
Androstenedione	A4	C9111	1/6000
Testosterone	T	R156	1/8000
Estrone sulphate	E1SO4	R522-2	1/12,000
17β-oestradiol	E2	C6E91	1/4000
Pregnenolone	P5	DE4170	
Dehydroandrosterone	DHEA	DEH3344	
Dehydrotestosterone	DHT	DE2330	

**Table 2:** Percentage of mice that developed metastasis in control and treated groups of IPC-366 and SUM149. * denoted significant differences between control and treatments.

% Mice with Metastasis	Control	Letrozole			STX-64		
		1 mg	5 mg	10 mg	1 mg	5 mg	10 mg
IPC-366	100%	10% *	10% *	10% *	0% *	0% *	0% *
SUM149	80%	20% *	20% *	20% *	0% *	0% *	0% *

## Data Availability

The data that support the findings of this study are available from the corresponding author upon reasonable request.

## References

[1] Krauss K., Stickeler E. (2020). Endocrine Therapy in Early Breast Cancer. Breast Care.

[2] McNamara K.M., Sasano H. (2015). The intracrinology of breast cancer. J. Steroid Biochem. Mol. Biol..

[3] Bulun S., Chen D., Moy I., Brooks D., Zhao H. (2012). Aromatase, breast cancer and obesity, a complex interaction. Trends Endocrionol. Metab..

[4] Manna S., Holz M.K. (2016). Tamoxifen Action in ER-Negative Breast Cancer. Signal Transduct. Insights.

[5] Gobbi S., Rampa A., Belluti F., Bisi A. (2014). Nonsteroidal aromatase inhibitors for the treatment of breast cancer: An update. Anticancer Agents Med. Chem..

[6] Sang X., Han H., Poirier D., Lin S. (2018). Steroid sulfatase inhibition success and limitation in breast cancer clinical assays: An underlying mechanism. J. Steroid Biochem. Mol. Biol..

[7] Chumsri S. (2015). Clinical utilities of aromatase inhibitors in breast cancer. Int. J. Womens Health.

[8] Foster P. (2021). Steroid Sulphatase and Its Inhibitors: Past, Present and Future. Molecules.

[9] Treeck O., Schüler-Toprak S., Ortmann O. (2020). Estrogen Actions in Triple-Negative Breast Cancer. Cells.

[10] Caceres S., Peña L., Silvan G., Illera M.J., Woodward W.A., Reuben J.M., Illera J.C. (2016). Steroid tumour environment in male and female mice model of canine and human inflammatory breast cancer. Biomed. Res. Int..

[11] Alonso-Diez A., Caceres S., Peña L., Crespo B., Illera J.C. (2021). Anti-Angiogenic Treatments Interact with Steroid Secretion in Inflammatory Breast Cancer Triple Negative Cell Lines. Cancers.

[12] Barkataki S., Javadekar M.J., Bradfield P., Murphy T., Witmer D.D., Van Golen K.L. (2018). Inflammatory Breast Cancer: A Panoramic Overview. J. Rare Dis. Res. Treat..

[13] Jansen M.P., Sas L., Sieuwerts A.M., Van Cauwenberghe C., Ramirez-Ardila D., Look M., Ruigrok-Ritstier K., Finetti P., Bertucci F., Timmermans M.M. (2015). Decreased expression of ABAT and STC2 hallmarks ER-positive inflammatory breast cancer and endocrine therapy resistance in advanced disease. Mol. Oncol..

[14] Hather G., Liu R., Bandi S., Mettetal J., Manfredi M., Shyu W.-C., Donelan J., Chakravarty A. (2014). Growth Rate Analysis and Efficient Experimental Design for Tumor Xenograft Studies. Cancer Inform..

[15] Purohit S.K., Chander L.W.L., Woo M.F.C., Parsons R., Jhalli B.V.L., Potter A., Reed M.J. (2008). Inhibition of Steroid Sulphatase Activity via the Percutaneous Route: A New Option for Breast Cancer Therapy. Anticancer Res..

[16] Rashid D., Pandam B.P., Vohora D. (2015). Reduced estradiol synthesis by letrozole, an aromatase inhibitor, is protective against development of pentylenetetrazole-induced kindling in mice. Neurochem. Int..

[17] Queiroga F., Pérez-Alenza M., Silvan G., Peña L., Lopes C., Illera J. (2005). Role of steroid hormones and prolactin in canine mammary cancer. J. Steroid Biochem. Mol. Biol..

[18] Illera J.C., Caceres S., Pena L., Monsalve B., Illera M.J., Woodward W.A., Reuben J.M., Silvan G. (2015). Steroid hormone secretion in inflammatory breast cancer cell lines. Horm. Mol. Biol. Clin. Investig..

[19] Peña L., Silván G., Pérez-Alenza M.D., Nieto A., Illera J.C. (2003). Steroid hormone profile of canine inflammatory mammary carcinoma: A preliminary study. J. Steroid Biochem. Mol. Biol..

[20] Illera J.C., Pérez-Alenza M.D., Nieto A., Jiménez M.A., Silvan G., Dunner S., Peña L. (2006). Steroids and receptors in canine mammary cancer. Steroids.

[21] Heldring N., Pike A., Andersson S., Matthews J., Cheng G., Hartman J., Tujague M., Strom A., Treuter E., Warner M. (2007). Estrogen receptors: How do they signal and what are their targets. Physiol. Rev..

[22] Honma N., Saji S., Hirose M., Horiguchi S.I., Kuroi K., Hayashi S.I., Utsumi T., Harada N. (2011). Sex steroid hormones in pairs of tumour and serum from breast cancer patients and pathobiological role of androstene-3β, 17β-diol. Cancer Sci..

[23] Abul-Hajj Y.J., Iverson R., Kiang D.T. (1979). Metabolism of pregnenolone by human breast cancer. Evidence for 17α-hydroxylase and 17,20-lyase. Steroids.

[24] Shin Y.Y., Kang E.J., Jeong J.S., Kim M.J., Jung E.M., Jeung E.B., An B.S. (2019). Pregnenolone as a potential candidate for hormone therapy for female reproductive disorders targeting ERβ. Mol. Reprod. Dev..

[25] Caceres S., Monsalve B., Peña L., de Andres P.J., Alonso-Diez A., Illera M.J., Woodward W.A., Reuben J.M., Silvan G., Illera J.C. (2018). In vitro and in vivo effect of flutamide on steroid hormone secretion in canine and human inflammatory breast cancer cell lines. Vet. Comp. Oncol..

[26] McNamara K.M., Yoda T., Takagi K., Miki Y., Suzuki T., Sasano H. (2013). Androgen receptor in triple negative breast cancer. J. Steroid Biochem. Mol. Biol..

[27] Palma C., Criscuoli M., Lippi A., Muratori M., Mauro S., Maggi C.A. (2000). Effect of the aromatase inhibitor MEN 11066, on growth of two different MCF-7 sublines. Eur. J. Pharmacol..

[28] Selli C., Turnbull A.K., Pearce D.A., Li A., Fernando A., Wills J., Renshaw L., Thomas J.S., Dixon J.M., Sims A.H. (2019). Molecular changes during extended neoadjuvant letrozole treatment of breast cancer: Distinguishing acquired resistance from dormant tumours. Breast Cancer Res..

[29] McNamara K.M., Guestini F., Sauer T., Touma J., Bukhlom I.R., Lindstrom J.C., Sasano H., Geisler J. (2018). In breast cancer subtypes steroid sulfatase (STS) is associated with less aggressive tumour characteristics. Br. J. Cancer.

[30] McNamara K.M., Yoda T., Nurani A.M., Shibahara Y., Miki Y., Wang L., Nakamura Y., Suzuki K., Yang Y., Abe E. (2014). Androgenic pathways in the progression of triple-negative breast carcinoma: A comparison between aggressive and non-aggressive subtypes. Breast Cancer Res. Treat..

[31] Higuchi T., Endo M., Hanamura T., Gohno T., Niwa T., Yamaguchi Y., Horiguchi J., Hayashi S.I. (2016). Contribution of Estrone Sulfate to Cell Proliferation in Aromatase Inhibitor (AI)-Resistant, Hormone Receptor-Positive Breast Cancer. PLoS ONE.

[32] Michmerhuizen A.R., Spratt D.E., Pierce L.J., Speers C.W. (2020). ARe we there yet? Understanding androgen receptor signaling in breast cancer. Breast Cancer.

[33] Dave N., Chow L., Gudelsky G., LaSance K., Qi X., Desai P. (2015). Preclinical pharmacological evaluation of letrozole as a novel treatment for gliomas. Mol. Cancer Ther..

[34] Foster P.A., Chander S.K., Parsons M.F.C., Newman S.P., Lawrance Woo L.W., Potter B.L.V., Reed M.J., Purohit A. (2008). Efficacy of three potent steroid sulfatase inhibitors: Pre-Clinical investigations for their use in the treatment of hormone-dependent breast cancer. Breast Cancer Res. Treat..

[35] Trabert B., Sherman M.E., Kannan N., Stanczyk F.Z. (2020). Progesterone and Breast Cancer. Endocr. Rev..

[36] Takagi K., Miki Y., Nagasaki S., Hirakawa H., Onodera Y., Akahira J., Ishida T., Watanabe M., Kimijima I., Hayashi S.I. (2010). Increased intratumoral androgens in human breast carcinoma following aromatase inhibitor exemestane treatment. Endocr. Relat. Cancer.

[37] Yue W., Wang J.P., Li Y., Fan P., Liu G., Zhang N., Conaway M., Wang H., Korach K.S., Bocchinfuso W. (2010). Effects of estrogen on breast cancer development: Role of estrogen receptor independent mechanisms. Int. J. Cancer.

[38] Wiebe J.P. (2006). Progesterone metabolites in breast cancer. Endocr. Relat. Cancer.

